# Preventive strategies against brain metastases: current state of the art and future directions

**DOI:** 10.1186/s42466-026-00467-7

**Published:** 2026-04-02

**Authors:** Franziska Maria Ippen, Franz Felix Konen, Peter Hau, Frank Winkler

**Affiliations:** 1 Department of Neurology, University Hospital Heidelberg, Im Neuenheimer Feld 400, 69120 Heidelberg, Germany; 2 National Center for Tumor Diseases (NCT), NCT Heidelberg, a partnership between DKFZ and University Hospital Heidelberg, 69120 Heidelberg, Germany; 3 Department of Neurology, Hannover Medical School, Carl-Neuberg-Str. 1, 30625 Hannover, Germany; 4 Department of Neurology and Wilhelm Sander-NeuroOncology Unit, Regensburg University Hospital, Franz-Josef-Strauß-Allee 11, 93053 Regensburg, Germany; 5 German Cancer Research Center (DKFZ), Clinical Cooperation Unit Neurooncology, 69120 Heidelberg, Germany

**Keywords:** Brain metastases, Prevention, Preventive strategies, Cancer neuroscience

## Abstract

**Background:**

Brain metastases (BM) represent a deleterious complication in many solid tumors. Despite advances in systemic therapy, BM are still associated with significant morbidity and mortality. Consequently, preventive strategies are gaining importance and represent a dynamic area of investigation.

**Methods:**

Relevant literature and clinical trials were included, focusing on systemic therapies, multimodal treatment approaches, and future therapeutic avenues. Clinical trials reporting on secondary (preventing BM in a yet BM-free cancer patient) and tertiary prevention (preventing formation of new and recurrence of existent BM in a patient who has already developed BM) were included.

**Results:**

A number of BM treatment-effective drugs have shown BM-preventive effects: In NSCLC, the EGFR-inhibitor osimertinib and ALK inhibitors like alectinib, brigatinib and lorlatinib lower the incidence of BM. In HER2-positive breast cancer, tucatinib and trastuzumab deruxtecan have preventive activity in patients without BM at baseline. In melanoma, BRAF/MEK inhibition and immunotherapy might be able to delay the onset of BM, although prospective data remains limited to date. Notably, drugs effective in BM therapy do not necessarily work in BM prevention, and vice versa. Preclinical data, including insights from Cancer Neuroscience, reveal novel preventive strategies warranting further exploration.

**Conclusions:**

Preventive strategies against BM in solid tumors are feasible, though data are sparse at this time. Preventing BM requires a personalized, multidisciplinary approach, integrating tumor biology, individual risk assessment, and therapeutic advances. Secondary prevention in patients with BM is as vital as tertiary prevention, warranting prospective validation of preclinical concepts.

## Background

Brain metastases (BM) account for the most common intracranial tumors in adult cancer patients and most commonly arise from lung cancer, breast cancer, and melanoma [1–3]. The incidence of BM is expected to rise, mainly due to prolonged survival of affected patients as systemic cancer treatments continue to improve and more detailed imaging techniques are becoming increasingly available. In addition, the pathobiology of metastatic lesions is brain-specific, partly due to a unique immuno-microenvironment and the blood-brain barrier (BBB) penetration of various systemic treatments is limited [1, 4].

Despite multimodal treatment approaches, which often include a combination of surgery, radiotherapy, chemotherapy, targeted agents and immunotherapy, BM are associated with a significant morbidity and mortality for affected patients [1, 5, 6]. This clinical burden highlights the need for effective therapeutic strategies, with an emphasis on prevention in order to delay or avoid metastatic spread to the central nervous system (CNS) altogether (secondary prevention) and disease recurrence after initial therapy (tertiary prevention).

Preventive strategies overall are gaining increasing importance in the management of cancer patients. Thus, the World Health Organization (WHO) underscores prevention as a cornerstone in global cancer control [7] and in Germany, the national “Decade Against Cancer” initiative (2019–2029) has emphasized translational and clinical efforts towards an effective, evidence-based cancer prevention [8]. In order to improve treatment options for patients with BM, especially with respect to preventive options, it is of utmost importance to increase our biological understanding of the metastatic process and molecular mechanisms that might drive BM and contribute to their growth. Most systemic cancer therapies are designed to target the primary tumor rather than metastases, highlighting the need for redesigned clinical trials and secondary prevention strategies that focus on preventing BM [9]. Furthermore, the emerging field of cancer neuroscience, exploring tumor–neuronal and microenvironmental interactions, offers promising avenues for innovative BM prevention strategies.

This comprehensive review aims at examining the evolving landscape of BM prevention in lung cancer, breast cancer, and melanoma. We present current secondary and tertiary preventive treatment approaches and outline ongoing trials and future therapeutic directions, driven by insights from cancer neuroscience and other preclinical areas.

## Methods

A comprehensive literature search was performed using PubMed, MEDLINE, and Embase databases for articles published between January 2014 (after the last clinical guideline for brain metastases from the Germany Society of Neurology, DGN was published) and July 2025. We included peer-reviewed clinical trials, meta-analyses, systematic reviews, guideline publications, expert consensus and translational studies focusing on prevention strategies for patients with brain metastases from the entities described above. Articles in English and German were included, articles lacking full-text availability were excluded. Publications on secondary prevention (avoiding CNS involvement in high-risk patients) as well as tertiary prevention (delaying CNS recurrence or progression) were considered. Studies were selected based on their relevance to lung cancer, breast cancer, or melanoma specifically, and those providing mechanistic or therapeutic insights from the field of cancer neuroscience were prioritized where appropriate.

In order to capture current developments on secondary and tertiary developments in patients with BM, registered ongoing clinical trials were also reviewed via ClinicalTrials.gov and the EU Clinical Trials Register. These included trials investigating novel CNS-directed agents and optimized local and systemic therapy regimens with CNS activity. Studies with an unknown status of more than 5 years and studies exclusively focusing on dietary supplements were excluded.

## Results

### General management recommendations of patients with brain metastases

According to the most recent interdisciplinary ASCO-SNO-ASTRO [10] and EANO-ESMO clinical practice guidelines [11], local therapies like surgical resection and radiation therapy mainly have a role in tertiary prevention, improving local control in patients with already existing brain metastases.

### Resection

According to the most recent interdisciplinary ASCO-SNO-ASTRO [10] and EANO-ESMO clinical practice guidelines [11], surgical intervention should be considered for patients with BM if there is need for histopathological diagnosis (e.g. in cases without a known primary malignancy) and in the presence of large tumors causing significant mass effect and clinical symptoms [10, 12–14]. However, the benefit of surgery in tertiary prevention is limited in patients with multiple brain metastases or uncontrolled systemic disease, except when the burden of remaining disease can be potentially controlled by other therapeutic options [10].

In patients with symptomatic BM, local therapies such as surgical resection, stereotactic radiosurgery (SRS) and radiotherapy should be offered irrespective of systemic treatment [10, 11]. In patients with asymptomatic BM, local therapy can be postponed if there are clear guideline recommendations on systemic treatment options which have shown to delay time to intracranial progression [10, 11].

### Radiotherapy

With respect to secondary prevention, prophylactic cranial irradiation (PCI) has been proven to be effective in reducing the incidence of BM in small cell carcinoma (SCLC), with reported risk reduction rates of 45–96% (RR 0.04–0.73) [15, 16]. Meta-analyses have furthermore shown that PCI in SCLC patients not only reduces the risk of BM, but also improves overall survival (OS), particularly in patients with radiologically confirmed BM [15, 16]. However, neurocognitive decline is a major concern in the application of PCI [17, 18]. Therefore, strategies investigating hippocampal-sparing WBRT options are currently under investigation: The randomized phase II/III NRG-CC003 trial (NCT02635009) has recently demonstrated to be as effective as standard PCI in preventing brain relapse, while lowering the risk of neurocognitive side effects and similar survival outcomes [19].

In NSCLC patients, several large trials as well as meta-analyses have confirmed a lower incidence of BM in patients treated with PCI [20–22]. However, no survival advantage could be demonstrated to date [20–22]. Therefore, current guidelines do not recommend PCI in NSCLC patients.

In the context of tertiary prevention, in patients with one up to four unresected BM, SRS alone should be considered opposed to WBRT/WBRT + SRS in BM < 3–4 cm in diameter, as investigated in trials which did not include radioprotectant strategies like hippocampal avoidance or the use of memantine. This recommendation excludes small-cell carcinoma [10, 11]. SRS alone should be pursued in patients with 1–2 resected BM if the surgical cavity is safely targetable and the remaining intracranial disease burden is taken into account [23]. Patients with either > 4 unresected or > 2 resected BM and a KPI ≥ 70, can be treated with SRS, WBRT, or a combination of WBRT+SRS [10, 11, 24]. In patients with a favorable prognosis or those receiving CNS-active systemic therapies, SRS should be preferred [10, 11]. In patients receiving WBRT, hippocampal avoidance should be offered whenever feasible and survival is estimated to exceed 4 months [10, 11, 25].

Of note, radiation sensitizers are not recommended and the optimal order of surgery and radiation therapy is still a matter of ongoing investigation [10, 11].

### Secondary and tertiary preventive strategies in lung cancer brain metastases

In NSCLC, the likelihood of developing BM and the designated secondary and tertiary prevention options largely depend on the underlying molecular alteration in the primary tumor (Table 1) [17, 26–29]. In contrast, small cell lung cancer (SCLC) shows a markedly high risk for developing BM (~ 50%) [17]. However, distinct molecular subtypes predictive of BM still remain to be defined in SCLC [17].

**Table 1:** Molecular alterations associated with NSCLC brain metastases: Incidence, risk for development of brain metastases and potential therapeutic implications

Molecular alteration	Incidence among NSCLC patients	Risk for the development of BM	Potential Therapeutic Agents
EGFR mutations (most frequently exon 19 deletions or L858R substitutions)	10–50%	10–50%	Osimertinib, erlotinib, gefitinib, afatinib, dacomitinib, amivantamab (exon 20 insertation mutations)
ALK rearrangements	7%	Up to 40%	Alectinib, ceritinib, brigatinib, lorlatinib, crizotinib
KRAS mutations	20–25%	Up to 40%	G12C mutations: Sotorasib, adagrasib
BRAF V600E mutations	1–2%	9–30%	Dabrafenib+trametinib
MET exon 14 skip mutations/high-level amplification	2–4%	Up to 36%	Capmatinib, tepotinib, crizotinib
ROS1 rearrangements	1–2%	Up to 30%	Entrectinib, crizotinib, lorlatinib, ceritinib
RET rearrangements	1–2%	Up to 30%	Selpercatinib, pralsetinib
NTRK rearrangements	0–1%	Up to 50%	Larotrectinib, entrectinib
HER2 Exon 20 insertion mutations	1–4%	6–30%	Trastuzumab, afatinib, ado-trastuzumab emtamsine, trastuzumab deruxtecan
PD-L1	≥ 1%: 14–76% ≥ 5%: 24–60% ≥ 50%: 4–43%	20–30%	Pembrolizumab, atezolizumab, nivolumab, tislelizumab

### EGFR Inhibition

Most EGFR mutations in NSCLC are exon 19 deletions or L858R (exon 21) mutations [26].

Regarding secondary prevention, first-line EGFR inhibition with the third-generation EGFR-inhibitor Osimertinib reduced the incidence of new brain metastases in NSCLC patients without baseline CNS involvement in the phase III FLAURA trial [30] (22% vs. 41% with standard EGFR TKIs).

With respect to tertiary prevention, patients with EGFR mutations treated with first- and second-generation tyrosine kinase inhibitors (TKIs) against EGFR like erlotinib, gefitinib or afatinib have shown intracranial response rates ranging from 70–88% [31, 32, 33]. However, the third generation EGFR TKI Osimertinib is the current standard of care in EGFR-mutant NSCLC with brain metastases. In the FLAURA-trial, Osimertinib has shown superior OS and PFS as well as intracranial response rates of 91% vs. 68%, when compared to earlier generation TKIs targeting EGFR [30], while in FLAURA2, osimertinib + platinum-pemetrexed was superior to treatment with Osimertinib alone [34]. Of note, patients in these trials were included if they had either asymptomatic/stable or previously irradiated BM^30,34–37^. Furthermore, Osimertinib has been shown to achieve intracranial responses in patients previously being treated with other EGFR-directed TKIs and developed a T790M resistance mutation [35, 36]. In patients with common (exon 19 deletion or L858R mutations) and uncommon (exon 20 insertions) EGFR mutations, amivantanab has furthermore shown promising results [38, 39]. The dual HER2 and EGFR inhibitor neratinib might have antitumor activity in selected patients with BM from EGFR-positive NSCLC, as results from the SUMMIT basket trial have shown [40].

### ALK Inhibition

In the context of secondary prevention, first-line treatment with next-generation ALK inhibitors has significantly reduced the incidence of new BM in patients without baseline intracranial tumor burden. In the phase III ALEX and J-ALEX trials [41, 42], NSCLC patients treated with the highly selective second-generation ALK TKI alectinib had a 12-month incidence of CNS of 9.4% compared to 41.4% with crizotinib (ALK/ROS1/MET TKI, first generation ALK inhibitor). In the phase III ALTA-1 L trial [43], brigatinib (second-generation ALK TKI with activity in crizotinib-resistant NSCLC), demonstrated a lower rate of intracranial disease progression as first site of progression (1% vs. 5% with crizotinib) in patients without BM at baseline. In the phase III CROWN trial [44], treatment with the third-generation ALK inhibitor lorlatinib vs. crizotinib resulted in an incidence of newly diagnosed BM of 3% vs. 33% in patients without BM at baseline, respectively over the course of treatment.

Addressing tertiary prevention, among ALK-directed targeted treatments, crizotinib improved intracranial disease control vs. chemotherapy in the PROFILE1014 phase III trial [45], but carries the risk of poor overall CNS penetration and p-glycoprotein efflux [17]. In line with this, alectinib showed superior CNS outcomes when compared to treatment with either crizotinib or chemotherapy in various phase II and III trials with respect to progression of BM [41, 42, 46, 47]. Similarly, brigantinib has shown reduced intracranial disease progression rates when compared to crizotinib in the ALTA phase II and ALTA-1 L phase III trial [43, 48]. The selective ALK TKI ceritinib demonstrated promising intracranial activity in the ASCEND-1/-2 and − 4 trials, assessing ceritinib in patients with asymptomatic and stable BM on prior exposure to ALK inhibitors or compared to crizotinib/chemotherapy [47, 48, 49]. Lorlatinib moreover has shown superior response rates over crizotinib and even remains effective after progression on second-generation ALK TKIs [50, 51]. However, its side effects and its possible application in later stages of therapy limit front-line use [26].

### Targeting KRAS, NTRK and ROS1 alterations

With respect to secondary preventive strategies, available data on KRAS G12C, NTRK and ROS1 inhibition are limited to date and do not allow for definite conclusions on a potential secondary preventive effect.

Regarding tertiary prevention, KRAS G12C-inhibition, sotorasib demonstrated a longer BM recurrence time vs. docetaxel (15.8 vs. 10.5 months; HR 0.52) in pretreated NSCLC with prior CNS disease (CodeBreaK200 phase III trial) [52], although these results did not reach statistical significance. Furthermore, a median intracranial response duration of 18.6 months was achieved by the CNS-penetrant KRAS G12C inhibitor adagrasib in treated and neurologically stable NSCLC BM previously treated with chemotherapy and immunotherapyin the KRYSTAL-1 trial [53].

With respect to both NTRK and ROS1 inhibition, the TKI entrectinib has shown promising CNS activity with complete intracranial response rates up to 35% in the STARTRK‑1/STARTRK‑2 and ALKA‑372‑001 trials in asymtomatic or stable and pretreated BM with these respective molecular alterations [54, 55].

### Targeting RET fusions, HER2-mutations and MET exon 14 skipping

Regarding secondary prevention in NSCLC patients with RET fusions, a pooled analysis of the phase II LIBRETTO-001 and LIBRETTO-321 trials [56, 57] showed that the RET-directed TKI selpercatinib in patients without BM at baseline exhibited no CNS progression for the duration of therapy. Although data on secondary prevention is limited, its favorable anti-tumor activity within the CNS suggests a potential to delay or prevent CNS involvement.

With respect to tertiary prevention and patients with RET fusion–positive advanced NSCLC and asymptomatic BM, selpercatinib revealed in the same pooled analysis intracranial complete response rates ranging from 23–38% [56, 57].

A pooled analysis from the phase II DESTINY-Lung01 and Lung02 trials, the antibody drug conjugate (ADC) trastuzumab deruxtecan (T-DXd) was evaluated in patients with stable HER2-mutant NSCLC BM [58]. At a dose of 5.4 mg/kg (DESTINY-Lung02), the median intracranial PFS (iPFS) was 9.2 months, with an intracranial overal response rate (iORR) of 12.5%. At a dose of 6.4 mg/kg (pooled results from DESTINY Lung01/Lung02), median iPFS was 4.4 months, with an iORR of 1.9% [58].

In patients with MET exon 14 skipping NSCLC and asymptomatic, pretreated BM, the phase II VISION trial (Cohort A) trial reported on the TKI tepotinib, achieving a median iPFS of 9.5 months, with an iORR of 39% [59]. The selective MET inhibitor capmatinib was investigated in a small number of patients with NSCLC BM in the GEOMETRY trial [60], achieving overall disease control in 92.3% of patients, while CNS progression was not reported.

### Immunotherapies

Secondary preventive effects of immunotherapies in NSCLC patients have mainly been examined via post-hoc analyses of existing trials: Both a post-hoc analysis of the CheckMate 227 trial comparing treatment with nivolumab plus ipilimumab vs. chemotherapy [61] and a pooled assessment of the KEYNOTE-001/-010/-024 and − 042 trials [62] for pembrolizumab in patients with PD-L1-positive NSCLC showed a survival advantage for patients with and without brain metastases, but did not specifically address the assessment of new developed BM. A recent meta-analysis suggests that anti-PD-1/PD-L1 blockade may delay the development of BM in NSCLC patients [63], but prospective data analyzing this topic specifically is lacking.

In contrast, with an emphasis on tertiary prevention, PD-1/PD-L1 inhibition has been evaluated throughout several phase II and III trials in NSCLC patients with BM, particularly in asymptomatic and pretreated cases. In the Atezo-brain trial, atezolizumab plus chemotherapy achieved an iORR of 42.7% with a median iPFS of 6.9 months [64]. Similarly, tislelizumab combined with chemotherapy showed an iORR of 46.7% [65]. In a recent phase II study, pembrolizumab monotherapy demonstrated intracranial responses in ~ 30% of patients with PD-L1-positive NSCLC and untreated, asymptomatic BM, with a median duration of 6 months in responders [66]. The combination of nivolumab with SRS in a recent phase II trial yielded an iPFS of 45.2%, although detailed response categorization was not provided in detail [67].

Phase III trials such as CheckMate 227 [61] and a pooled analysis of the KEYNOTE trials − 001, 010, 024, and 042 [62] included patients with treated or stable BM and showed survival benefits for PD-1/PD-L1 inhibitors over chemotherapy in these subgroups. However, detailed intracranial response data or median intracranial response duration was not reported.

### Secondary and tertiary preventive strategies in breast cancer brain metastases

In breast cancer, systemic therapy options for secondary and tertiary prevention in patients with BM vary by hormone receptor status[26] (Table 2).

**Table 2:** Molecular alterations associated with breast cancer brain metastases: Incidence, risk for development of brain metastases and potential therapeutic implications

Hormone receptor (HR) subtype	Incidence among breast cancer patients	Risk for the development of BM	Potential Therapies
HER2 positive/ HR-negative	6–11%	26–40%	Trastuzumab + pertuzumab + paclitaxel/docetaxel; high dose trastuzumab/pertuzumab; T-DM1; lapatinib + capecitabine; tucatinib + trastuzumab + capecitabine, trastuzumab deruxtecan (T-DXd)
HR‑positive / HER2‑negative (Luminal A)	Ca. 70%	7–12%	Abemaciclib, alpelisib/fulvestrant
HR‑positive / HER2‑positive (Luminal B)	Ca. 10%	24–30%	Abemaciclib
Triple‑negative (HR‑negative / HER2‑negative, TNBC)	Ca. 11%	Ca. 25%	Sacituzumab govitecan

### HER2-positive breast cancer

With regard to secondary prevention, recent prospective data suggest that HER2-targeting agents like tucatinib [68] and trastuzumab-deruxtecan [69] may delay and/or reduce the incidence BM in patients without baseline BM, although formal phase II and III trials with explicit endpoints addressing this subject in BM-naïve breast cancer patients are limited: Current post-hoc analyses from HER2CLIMB indicate a 45% reduction in risk of developing new BM in patients without BM at baseline when tucatinib was added to trastuzumab/capecitabine.

In HER2-positive patients with BM with respect to tertiary prevention, the combination of trastuzumab/pertuzumab plus paclitaxel/docetaxel is characterized by its poor CNS penetration [26]. The PATRICIA trial assessed patients with BM on high-dose trastuzumab/pertuzumab who had progressed on prior trastuzumab and radiotherapy and demonstrated a intracranial response rate of 11% [70]. Subsequently, the ADC trastuzumab emtansine (T-DM1) was investigated in the single-arm phase IIIb KAMILLA trial in breast cancer BM post-radiation and radiation-naïve patients and yielded CNS-responses in 33% and 49%, respectively [71]. The phase II LANDSCAPE trial evaluated the dual TKI lapatinib in combination with capecitabine in patients with BM who were again post-radiation and radiation-naïve [72]. CNS responses were 66% in radiation-naïve and ~ 20% in post-radiation patients [72]. The irreversible pan-HER TKI neratinib in combination with capecitabine was evaluated in the phase II trial TBCRC 022 and revealed CNS response rates of 33–49%, depending on prior lapatinib use [73]. The HER2CLIMB trial assessed adding tucatinib (a more selective HER2-targeting TKI) vs. placebo to the combination of trastuzumab and capecitabine in HER2-positive breast cancer patients with BM [68]. The confirmed iORR was more than doubled with tucatinib (47.3% vs. 20.0%), making HER2CLIMB the first randomized trial showing improved intracranial antitumor activity in this patient population [68]. With respect to the ADC trastuzumab deruxtecan (T-DXd) in HER2-positive breast cancer BM, several phase II and III trials have demonstrated robust intracranial efficacy: In the TUXEDO-1 phase II trial, T-DXd achieved an iORR of 73.3% in patients with active or progressing BM [74]. The phase III trials DESTINY-Breast03 and DESTINY-Breast12 further confirmed these findings in larger cohorts, demonstrating superior CNS PFS and intracranial responses compared to the ADC T-DM1, including patients with active or stable BM [69,75].

### Hormone receptor-positive, HER2-negative breast cancer

Secondary as well as tertiary preventive treatment options for patients with hormone receptor-positive, HER2-negative breast cancer BM are limited: With respect to tertiary prevention, CDK4/6 inhibitors combined with hormonal therapy are standard in metastatic disease, but intracranial responses are low; a phase II trial of abemaciclib showed a response rate of 5% in hormone receptor positive HER2-negative and 0% in hormone receptor positive HER2-positive patients, with a clinical benefit rate ranging from 11–24% [76]. The PI3K-inhibitor alpelisib may offer intracranial activity in patients with *PIK3CA* mutations, highlighting the potential value of molecular profiling in affected patients [77]. Across all subtypes of breast cancer, patients with BM and BRCA mutations may benefit from PARP inhibitors, but prospective data on intracranial efficacy is still lacking. Although a combined approach of carboplatin with bevacizumab may hold promise [78], bevacizumab to date lacks FDA approval for metastatic breast cancer in the U.S. Therefore, local therapies remain an essential cornerstone of BM treatment, and improved systemic treatments with CNS activity are urgently needed, especially in TNBC and HR+/HER2 − subtypes.

### Triple-negative breast cancer

To date, secondary preventive strategies for TNBC are largely unexplored in this population.

Moreover, only few trials have evaluated tertiary preventive systemic treatment options for patients with tripe-negative breast cancer (TNBC). Of those, the ASCENT trial, assessing the ADC sacituzumab govitecan yielded longer PFS in patients with stable BM (2.8 vs. 1.6 months, HR 0.682, 95% CI 0.379–1.228) compared to treatment of physician’s choice, while OS was similar between the two groups (7.0 vs. 7.5 months, HR 0.956, 95% CI 0.546–1.675) [79]. Immunotherapies like atezolizumab, often used in combination with nab-paclitaxel or carboplatin/gemcitabine in PD-L1–positive TNBC, may offer potential benefit against BM [26]. However, the current clinical evidence is limited.

### Secondary and tertiary preventive strategies in melanoma brain metastases

In BRAF-mutant melanoma patients without baseline BM, first-line immunotherapy, alone or in combination with BRAF-targeted therapies, seems to delay the onset of BM [80, 81], although clinical data on this topic is sparse.

Systemic therapies with respect to tertiary prevention in melanoma BM have significantly led to improved patient outcomes and durable CNS responses in recent years, mainly due to immunotherapies and BRAF-targeted treatment approaches (Table 3) [26].

**Table 3:** Molecular alterations associated with melanoma brain metastases: Incidence, risk for development of brain metastases and potential therapeutic implications

Molecular alteration	Incidence among melanoma patients	Risk for the development of BM	Potential Therapies
BRAF V600E mutations	Ca. 40–50%	Ca. 40–50%	Dabrafenib + trametinib (BRAF + MEK); vemurafenib + cobimetinib, encorafenib + binimetinib, trametinib, cobimetinib, binimetinib trametinib, cobimetinib, binimetinib, and in combination with immune checkpoint inhibitors
NRAS mutations	Ca. 15–25%	18%	Binimetinib
CDKN2A alteration	Ca. 15–40%	No clear evidence available	Palbociclib, abemaciclib, ribociclib
PIK3CA mutation	Ca. 2.5%	No clear evidence available	Buparlisib
KIT	Ca. 3–28%	< 10%	Sunitinib, nilotinib, imatinib, sorafenib
PD-L1 expression (tumor/immune cells)	Ca. 30%	No clear evidence available	Nivolumab, pembrolizumab, sometimes in combination with CTLA-4 inhibitors (ipilimumab, etc.)

To date, the mainstay of BRAF/MEK-inhibition in BRAFV600-mutant melanoma BM remains a combination of dabrafenib plus trametinib according to the phase II COMBI-MB trial [82] with impressive intracranial response rates of 44–59% in affected patients. Other BRAF/MEK combinations mostly are lacking prospective data on their efficacy in patients with BM, but are a feasible therapeutic option [26]. Limited intracranial responses have been observed in patients with NRAS mutations and BM treated with binimetinib [83]. Immunotherapy is preferred as a first-line treatment option for BRAF wildtype or stable BRAF-mutant patients. The current standard regimen for melanoma patients with BM involves a combination of concurrent ipilimumab plus nivolumab, followed by maintenance nivolumab. The key clinical trials leading to this approach were the phase II trials CheckMate 204 and ABC, demonstrating intracranial response rates of up to 60% in asymptomatic and up to 17% in symptomatic, steroid-dependent patients [84, 85]. The addition of radiotherapy may lead to synergistic effects with immunotherapy [26]. However, the currently available evidence on this topic is mainly based on retrospective studies.

### Landscape of clinical trials focusing on preventive strategies

In total, we identified 13 trials selectively focusing on secondary and tertiary preventive strategies in patients with brain metastases. The identified studies were further categorized according to intervention type, recruitment status, study status and location (Table 4).

**Table 4:** Ongoing clinical trials with a primary focus on preventive strategies for brain metastases

NCT -identifier	Title	Study status	Conditions	Interventions	Phase	Location/ Centers
NCT04891471	WHOle Brain Irradiation or STEreotactic Radiosurgery for Five or More Brain Metastases (WHOBI-STER) (WHOBI-STER)	recruting	Brain metastases	Radiation: Stereotactic RadioTherapy; Radiation: Whole Brain Irradiation	na	Italy
NCT03028337	Single Versus Multifraction Salvage Spine Stereotactic Radiosurgery for Previously Irradiated Spinal Metastases	recruting	Solid tumor malignancy and radiographic evidence of spine metastasis	Radiation: Spine Radiosurgery; Behavioral: Questionnaires	2	Houston, Texas, US
NCT04099251	A Phase 3, Randomized, Double-Blind Study of Adjuvant Immunotherapy with Nivolumab versus Placebo after Complete Resection of Stage IIB/C Melanoma (CheckMate 76 K: CHECKpoint pathway and nivoluMAb clinical Trial Evaluation 76 K)	active, not recruting	Completely resected Stage IIb/c melanoma patients	Drug: Nivolumab	3	worldwide
NCT05323955	Secondary BRain Metastases Prevention After Isolated Intracranial Progression on Trastuzumab/​Pertuzumab or T-DM1 in Patients With aDvanced Human Epidermal Growth Factor Receptor 2 + brEast Cancer With the Addition of Tucatinib (BRIDGET)	active, not recruiting	HER2 + BC by ASCO-CAP guidelines	Drug: Trastuzumab; Drug: Trastuzumab Emtansine (T-DM1); Drug: Pertuzumab; Drug: Tucatinib	2	San Francisco, California, US
NCT02831959	Pivotal, Open-label, Randomized Study of Radiosurgery With or Without Tumor Treating Fields (TTFields) for 1–10 Brain Metastases From Non-small Cell Lung Cancer (NSCLC).	completed	Histologically or cytologically confirmed primary or metastatic NSCLC tumor	Device: NovoTTF-200 M device; Other: Best Standard of Care	3	worldwide
NCT00639366	Radiation Therapy to the Head in Preventing Brain Metastases in Women Receiving Trastuzumab and Chemotherapy for Metastatic or Locally Advanced Breast Cancer	completed	Metastatic or locally advanced BC	Biological: trastuzumab; Drug: chemotherapy; Radiation: radiation therapy	3	UK
NCT02038647	A Randomized, Double-blind, Placebo-controlled, Phase 2 Clinical Trial of Alisertib (MLN8237) in Combination With Paclitaxel Versus Placebo in Combination With Paclitaxel as Second Line Therapy for Small Cell Lung Cancer (SCLC)	completed	SCLC	Drug: Alisertib (MLN8237)	2	worldwide
NCT03995667	Tumor Treating Fields (TTFields) Therapy to Manage Brain Metastases in Small Cell Lung Cancer	terminated (low accrual)	Brain metastases in SCLC	Other: Questionnaire Administration; Device: Tumor Treating Fields Therapy (Optune-Tumor Treating Fields (TTFields). Transducer arrays affixed to scalp, worn continuously.)	2	Phoenix, Arizona, US, Portland, Oregon, US
NCT00638963	Temozolomide as a Prophylaxis Against Brain Recurrence in Participants With Metastatic Breast Cancer (P05225 AM2) (STOP)	terminated (low accrual)	Metastatic BC	Drug: temozolomide	2	no location data
NCT01613482	TraStuzumAb-Radiotherapy : Impact on the Cerebral Prevention (TSARINE)	terminated (lack of enrollment)	Metastatic BC Expressing HER2/NEU	Radiation: cerebral prophylactic radiation; Drug: Trastuzumab; Drug: Other chemotherapy in association with Trastuzumab	3	France
NCT00745797	Prophylactic Cranial Irradiation (PCI) Versus no PCI in Non Small Cell Lung Cancer After a Response to Chemotherapy (PCI)	terminated (low accrual, no funding support)	NSCLC (brain-metastasis free)	Radiation: Radiotherapy	3	Guangzhou, Guangdong, China
NCT04856475	Evaluation of Neratinib for Treatment and Prevention of Subsequent CNS Event(s) in Patients With Brain Metastasis of Advanced HER2 Positive Breast Cancer (NeraBrain)	withdrawn (termination of collaboration)	Advanced HER2 Positive BC	Drug: Neratinib	2	France
NCT06676917	Datopotamab Deruxtecan (Dato-DXd) for Non-Small Cell Lung Cancer (NSCLC) Patients with Active Brain Metastases (TUXEDO-5)	withdrawn	Non-squamous NSCLC with active brain metastases (BMs)	Drug: Datopotamab deruxtecan	2	no location data

Medication-based strategies (*n* = 6) were primarily composed of evaluating targeted therapies, chemotherapies, and immunotherapies. Of those, a randomized, double-blind trial focusing on secondary prevention is currently assessing adjuvant immunotherapy with nivolumab vs. placebo after complete resection of stage IIB/C melanoma (CheckMate 76 K, NCT04099251, phase III, active, not recruiting). Tertiary preventive strategies include the evaluation of datopotamab deruxtecan (Dato-DXd) in NSCLC patients with active brain metastases (TUXEDO-5; NCT06676917; phase II, withdrawn); the addition of tucatinib after intracranial progression on anti-HER2 therapy with either trastuzumab/pertuzumab or T-DM1 (BRIDGET; NCT05323955; phase II, active, not recruting); alisertib in combination with paclitaxel vs. placebo in combination with paclitaxel as second line therapy for SCLC (NCT02038647, phase II, completed); and the use of temozolomide in patients with metastatic breast cancer (STOP, NCT00638963, phase II, terminated). A medication-based trial focusing on both secondary and tertiary prevention moreover investigated neratinib in advanced HER2 + breast cancer (NeraBrain; NCT04856475, phase II, withdrawn).

Radiation-based preventive strategies (*n* = 5) include PCI in NSCLC patients (NCT00745797, phase III, terminated) and PCI in combination with trastuzumab in patients with HER2 + breast cancer (TSARINE, NCT01613482, terminated; NCT00639366; phase III, completed) as secondary preventive treatment options. Tertiary preventive radiation-based strategies assessing WBRT vs. SRS for five or more BM (WHOBI-STER, NCT04891471, phase: not applicable, recruiting); and single vs. multi-fraction salvage spine SRS for previously irradiated spinal metastases (NCT03028337, phase II, recruiting).

Device-based preventive strategies (*n* = 2) aim at investigating tertiary preventive potential of Tumor Treating Fields (TTF) in patients with SCLC BM (NCT03995667, phase II, terminated) and TTF with or without SRS in patients with 1–10 NSCLC BM (NCT02831959, phase III, completed). At the time of data extraction, outcome data were not available for any of the identified studies.

### Promising preclinical strategies for the prevention of brain metastases: insights from cancer neuroscience and other fields

This overview underscores the fact that despite an increasing recognition, only a limited number of clinical trials are currently designed to address prevention in patients with BM as a primary objective. Especially trials dedicated to secondary prevention strategies for patients with BM remain a significant gap in neuro-oncology, because most trials to date rather focus on treatment of already existing BM rather than on strategies to prevent their development in the first place [9].

However, recent advances in the emerging field of Cancer Neuroscience have led to novel insights of the metastatic process and therefore, to the identification of novel preclinical strategies that may hold promise for the prevention of BM [86]. Amongst them, in vivo imaging studies have revolutionized the visualization of sequential steps of BM formation in real-time and led to establishing a foundational model to investigate early mechanisms and potential therapeutic interventions [87].

Recent work has highlighted the neurobiological processes supporting the progression of brain metastases: direct bona-fide glutamatergic neuron-to-cancer synapses of the AMPAR type have been identified in brain metastases from breast cancer and melanoma [88], and recently also NSCLC [86]. These synaptic interactions appear to have distinct roles across metastatic stages. While AMPAR-dependent synapses promote progression throughout the brain metastatic cascade, NMDA receptor stimulation of perisynaptically located cancer cells supported macrometastatic outgrowth in breast cancer models [89, 90]. Moreover, neurotransmitters like glutamate and GABA have been demonstrated to mediate the metastatic process to the brain at various stages [89]. It has recently been shown that SCLC cells exploit neuronal activity in the brain in order to stimulate tumor proliferation via glutamatergic and GABAergic neurons through paracrine signaling and direct neuron-to-cancer synapses [91]. These synapses induce SCLC cell depolarization and calcium influx, which promotes intracranial tumor growth [91]. Preclinical studies have further demonstrated that tumor cells from lung cancer, breast cancer and melanoma use peripheral nervous system (PNS) traits to metastasize to the brain. To date, it is a matter of ongoing investigation whether these features are acquired in the brain or are already present in a brain-tropic subpopulation of cells that subsequently metastasize to the brain [86]. Moreoever, astrocyte-to-tumor gap junctions, particularly via cGAMP transfer, have been associated with promoting brain metastasis [92]. Emerging evidence suggests that brain metastases form intrinsic tumor cell networks through gap junctions [93]. In in vivo studies, consecutive inhibition with brain-penetrant gap junction blockers resulted in reduced calcium signaling and decreased tumor burden [93]. These networks were exclusively composed of tumor cells, highlighting a selective therapeutic vulnerability in brain metastases [93].

All in all, these preclinical studies from the emerging area of Cancer Neuroscience point towards neuromodulatory inhibitors of neuro-cancer connectivity like AMPARs (e.g., with the approved antiepileptic drug perampanel [86]), or inhibitors of cancer-cancer connectivity like gap junction inhibitors (e.g., with the approved drug meclofenamate), as a novel class of promising agents that should be investigated in BM prevention clinical trials.

In addition, data from experimental breast cancer mouse models have demonstrated that temozolomide can profoundly prevent the outgrowth of BM in an MGMT‑dependent manner [94], providing a rationale for further prospective evaluation in a clinical trial. Furthermore, translational data on the role of vascular biology on BM formation indicate that early extravasation, contacts to microvessels and perivascular growth (melanoma) or early angiogenesis (lung cancer) as part of the BM forming process can be targeted with VEGF-A inhibition and therefore be a promising preventive strategy [87, 95]. However, no systemic extracranial preventive activity could be detected [95]. Recent studies have moreover identified endothelial remodeling and tumor-driven local blood coagulation as critical parts of the metastatic process, making inhibition of MMP9 [96] and von Willebrand factor (vWF) [97] a promising secondary preventive strategy. Beyond vascular mechanisms, preclinical in vivo studies have demonstrated that melanoma cells require early activation of the PI3K/Akt/mTOR pathway in order to form BM and that preventive inhibition of this pathway may reduce the incidence of BM [98].

In summary, these preclinical data highlight that targeting the cerebral microenvironment might be a potential route for BM prevention in addition to tumor cell directed therapies alone.

## Discussion

Prevention of BM is becoming increasingly important as systemic therapies in recent years have significantly improved patient survival and reveal the CNS as a common site of disease relapse [1, 2]. Despite advances in imaging and screening, trial design remains challenging. The incidence, development and response with respect to BM should therefore be reported whenever possible to enable an assessment of CNS preventive effects.

However, a major challenge lies in optimizing clinical trial design when secondary and tertiary preventive strategies in BM need to be assessed. This includes careful patient selection: Ideally, the approach should be based on predictive biomarkers, such as circulating tumor DNA and established brain-tropic gene signatures of the primary tumor to allow for an enrollment of high-risk patients without BM at baseline [9, 26]. An alternative endpoint to specifically address secondary prevention could be time to CNS metastasis in this setting rather than tumor shrinkage, as currently analyzed for tertiary preventive strategies [9]. Therefore, optimal secondary preventive therapeutic agents need to be well tolerated and able to cross the BBB with favorable metabolic stability within the brain, targeting metastatic colonization rather than tumor growth of already existing BM [9, 26].

With respect to tertiary preventive strategies, due to the encouraging CNS-activity of several systemic treatment options, the current ASCO-SNO-ASTRO guideline has proposed that local therapy may be postponed if brain metastases are asymptomatic and can be treated with the following systemic agents according to their respective molecular alterations [10]: (1) EGFR-mutant NSCLC: Osimertinib or icotinib; (2) ALK-rearranged NSCLC: Alectinib, brigatinib, or ceritinib; (3) PD-L1–positive NSCLC: Pembrolizumab with pemetrexed + platinum; (4) melanoma: Ipilimumab + nivolumab (all patients) or dabrafenib + trametinib (BRAF V600E); (5) HER2 + breast cancer: Tucatinib + trastuzumab + capecitabine after prior HER2 therapy. These decisions should always weigh risks and benefits and should be discussed in a multidisciplinary setting [10].

With respect to tertiary preventive strategies in patients with BM, several open questions should be addressed in future clinical trials: In NSCLC with systemically targetable alterations, the role of additional radiation therapy remains controversial. Some retrospective studies suggest a potential benefit from combining TKIs with CNS radiation therapy [26], but prospective data from ongoing randomized trials (NCT03769103, NCT04634110) are needed to address this question. In breast cancer, although there has been impressive progress with respect to systemic therapy options for patients with HER2-positive breast cancer with BM, the best sequential strategy as well as dual HER2-directed treatments are still a matter of ongoing research. Several clinical trials are currently evaluating new HER2-targeted combinations, including tucatinib with T-DM1 (NCT03975647), T-DM1 with neratinib (NCT01494662), and trastuzumab deruxtecan with tucatinib (NCT04539938). With respect to TNBC, an active SWOG trial is currently evaluating the CNS activity of sacituzumab govitecan specifically in patients with active BM (NCT04647916), a crucial step towards systemic treatment of BM that were not previously treated locally. In this setting, PD-L1 blockade in combination with bevacizumab and cisplatin/carboplatin is also currently under investigation in active TNBC BM and has yielded promising results in a preliminary analysis [99].

In patients with melanoma BM, ongoing trials aim at exploring the role of combining immunotherapies with VEGF inhibitors and BRAF/MEK-inhibition with immunotherapies (NCT04511013). New BRAF/MEK inhibitors with an improved CNS-penetration are equally under investigation as a next generation of these inhibitors (NCT04543188, NCT03332589, NCT04190628). Similar to NSCLC, trials investigating systemic therapies in combination with radiation are being investigated (NCT03340129).

Furthermore, immune-checkpoint blockade offers a promising strategy for patients across various histologies: A phase 2 trial of pembrolizumab in patients with untreated or progressive brain metastases from different primary tumors showed an intracranial benefit rate of 42.1% and a median overall survival of 8 months [100], suggesting that PD-1 blockade may benefit patients beyond melanoma and NSCLC, where these treatments have been extensively analyzed in the past. Although PARP-inhibitors have demonstrated systemic efficacy in BRCA-mutated and other tumors, their role in patients with BM remains a matter of ongoing investigation. To date, most trials analyzing PARP inhibitors either only included stable, previously treated BM and did not report dedicated CNS endpoints [101, 102], or focused on combined treatment approaches with chemotherapy [103] or radiotherapy [104] and provided modest results. Several phase II trials are now addressing this gap in the literature, including also active progressing BM (NCT05700721; niraparib + PD-1 inhibitor dostarlimab; STARLET trial) and reporting on the efficacy of combination therapies with defined intracranial endpoints (NCT04711824, PARP inhibitor + SRS; SOLARA trial).

Lastly, the emerging field of Cancer Neuroscience has led to an improved understanding of the metastatic process to the brain and how neurons influence the growth of BM via direct and indirect mechanisms, revealing potential targets for secondary preventive strategies. It will be crucial for future systemic treatment approaches to determine how the nervous system also influences immune responses, with neurons affecting microglia and T cell functions, impacting antitumor activity in BM and how neurotransmitter receptors like NMDAR, AMPAR, and GABA drive tumor growth of BM [86]. Understanding these mechanisms from cancer biology and how the crosstalk between tumor cells and the nervous system shapes the metastatic process to the brain will therefore be a crucial aspect for improving systemic treatment strategies- especially in the early stages, where prevention takes place.

## Conclusions

Brain metastases continue to represent a significant challenge in the management of adult cancer patients. While therapeutic advances in both systemic and local treatment approaches have improved patient outcomes in recent years, secondary and tertiary prevention strategies are now becoming increasingly important, mainly due to prolonged survival, improved screening options and the limited penetration and efficacy of various systemic treatments within the CNS. The emergence of Cancer Neuroscience has opened up new avenues to an improved understanding of the metastatic process to the brain. This development unfolds new promising investigative opportunities to tackle these particular targets in order to contribute to a secondary prevention of BM in the first place. After encouraging preclinical results, prospective clinical trials are currently planned to evaluate preventive interventions accordingly. In order to optimize future clinical trial designs for secondary preventive strategies, biomarker-guided patient selection, long-term safety monitoring of treatments, and endpoints rather focused on time to development of BM rather than tumor shrinkage should be implemented. Moreover, the incidence of BM should ideally be reported whenever possible, to capture potential preventive effects on the CNS. Overall, a multidisciplinary treatment approach integrating knowledge from (neuro)oncology and neurobiology will be crucial for the development of effective preventive treatment strategies in affected patients with BM.

## Data Availability

Not applicable.

## Abbreviations

ADC: Antibody–Drug Conjugate
ALK: Anaplastic Lymphoma Kinase
AMPAR: α-Amino-3-hydroxy-5-methyl-4-isoxazolepropionic Acid Receptor
ASCO: American Society of Clinical Oncology
BBB: Blood–Brain Barrier
BM: Brain Metastases
CDK4/6: Cyclin-Dependent Kinase 4/6
CNS: Central Nervous System
EGFR: Epidermal Growth Factor Receptor
EANO: European Association of Neuro-Oncology
ESMO: European Society for Medical Oncology
FDA: Food and Drug Administration
GABA: Gamma-Aminobutyric Acid
HER2: Human Epidermal Growth Factor Receptor 2
HR: Hazard Ratio
HR+: Hormone Receptor Positive
iORR: Intracranial Overall Response Rate
iPFS: Intracranial Progression-Free Survival
KPI: Karnofsky Performance Index
KRAS: Kirsten Rat Sarcoma Virus
MET: Mesenchymal–Epithelial Transition
MRI: Magnetic Resonance Imaging
MMP9: Matrix Metalloproteinase 9
NMDA: N-Methyl-D-Aspartate
NSCLC: Non–Small Cell Lung Cancer
NTRK: Neurotrophic Tyrosine Receptor Kinase
OS: Overall Survival
PARP: Poly (ADP-Ribose) Polymerase
PCI: Prophylactic Cranial Irradiation
PD-1: Programmed Cell Death Protein 1
PD-L1: Programmed Death-Ligand 1
PFS: Progression-Free Survival
PNS: Peripheral Nervous System
RET: Rearranged During Transfection
RANO-BM: Response Assessment in Neuro-Oncology Brain Metastases
ROS1: c-ros Oncogene 1
SCLC: Small Cell Lung Cancer
SNO: Society for Neuro-Oncology
SRS: Stereotactic Radiosurgery
T-DM1: Trastuzumab Emtansine
T-DXd: Trastuzumab Deruxtecan
TIL: Tumor-Infiltrating Lymphocyte
TKI: Tyrosine Kinase Inhibitor
TNBC: Triple-Negative Breast Cancer
TTF: Tumor Treating Fields
vWF: von Willebrand Factor
WBRT: Whole Brain Radiotherapy
WHO: World Health Organization
